# Investigating the basis for the antidepressant effects of *Gleditsiae spina* using an integrated metabolomic strategy

**DOI:** 10.22038/ijbms.2021.51975.11781

**Published:** 2021-04

**Authors:** Tong Liu, Ning Zhou, Yangang Cao, Ruihao Xu, Zhen Liu, Xiaoke Zheng, Weisheng Feng

**Affiliations:** 1 School of Pharmacy, Henan University of Chinese Medicine, Zhengzhou, China; 2 The Engineering and Technology Center for Chinese Medicine Development of Henan Province, Zhengzhou 450046, RP China

**Keywords:** Biomarker, Depression, Flavonoids, Metabolomics, Metabolic profiling

## Abstract

**Objective(s):**

*Gleditsiae spina *(GS) is a natural antidepressant but its mechanisms of action remain unclear. In the present study, taxifolin (Tax) was selected to determine the role of flavonoids in the antidepressant effects of GS.

**Materials and Methods:**

Urine samples from C57BL/6 mice were analyzed based on ultra performance liquid chromatography-quadrupole time of flight mass spectrometry (UPLC-Q/TOF-MS). Then, we investigated the therapeutic effects of GS and Tax in depression models *in vivo.* An integrated metabolomic approach was used to examine the metabolic profiles of GS/Tax groups and corticosterone model groups (Cor). Metabolic networks in response to GS/Tax treatment were established for the comparison of antidepressant activities.

**Results:**

Corticosterone exposure significantly increased serum levels of corticosterone but decreased serum levels of 5-hydroxytryptamine and sucrose consumption (*P*<0.01). Treatment with GS and Tax improved all measured variables compared to those of the corticosterone-exposed group (*P*< 0.01). The antidepressant effects of GS and Tax involved the regulation of pentose and glucuronate interconversions, arginine and proline metabolism, phenylalanine metabolism, taurine and hypotaurine metabolism, and the citrate cycle.

**Conclusion:**

These findings indicate that flavonoids form the pharmacodynamic basis of the antidepressant effects of GS. Moreover, our findings highlight that integrated metabolomics provides a powerful tool to study the mechanisms and material basis of Chinese herbs.

## Introduction

Depression is a common and serious medical illness (1). The etiology of depression is incompletely understood, but genetic, hormonal, immunological, and neuroendocrinological mechanisms play a role (2-4). Currently, tricyclic antidepressants, selective serotonin reuptake inhibitors (SSRIs), serotonin/ norepinephrine reuptake inhibitors, and monoamine oxidase inhibitors are the four major classes of antidepressants used clinically (5, 6). However, a number of adverse effects—including arrhythmias, dizziness, tremors, cognitive impairments, urinary retention, and sexual dysfunction—limit the use of these drugs. Alternative therapeutics have been studied, among which Chinese herbs have been favored due to their low levels of associated side effects (7, 8). *Gleditsiae spina *(GS), also known as Zao Jiao Ci (in Chinese), is an important herb with various medicinal properties including antitumor (9, 10), anti-inflammatory (11), and antiatherogenic effects (12). GS, the thorn of *Gleditsia sinensis* Lam, is rich in flavonoids (13, 14), particularly taxifolin (Tax), which is thought to form the basis of the pharmacological activities of GS. Tax exhibits antioxidant, anti-inflammatory, and antiglycation effects (15-17). In addition, Tax has pleiotropic neuroprotective effects, suggesting it has the potential to prevent or ameliorate depression (18, 19).

In the present study, we investigated the role of Tax as a component of GS in mediating the antidepressant effects of GS. Depression models *in vivo* were established using corticosterone. Physiological indexes—including serum 5-hydroxytryptamine, corticosterone, and sucrose consumption—were examined to assess the antidepressant effects of GS and Tax. A simultaneous metabolomic approach was used to compare the systemic effects of GS and Tax through metabolic networks. Integrated data showed that the flavonoids present in GS were responsible for GS-mediated antidepressant effects.

## Materials and Methods

***Chemicals and reagents***

Corticosterone and fluoxetine were purchased from Cayman (USA) and Patheon (France), respectively. GS was obtained from the Luoyang Production Base of Medicinal Materials (Henan, China) and was authenticated by Professor Sui-Qing Chen. Voucher specimens were deposited in our laboratory at the Henan University of Chinese Medicine. Tax was obtained from GS. All other chemicals were of LC-MS grade. Enzyme-linked immunosorbent assay (ELISA) kits were obtained from Suzhou Calvin Biotechnology Co., Ltd. (China).

***Extraction***

GS was decocted in water three times (10 l × 3, 1 hr each time) at 100 °C and combined supernatants were evaporated and dried in a vacuum to obtain GS extracts.

***Animal models***

Male C57BL/6 mice (weighing 18–22 g) were obtained from the Beijing Vital River Laboratory Animal Technology Co., Ltd. (animal license number: SCXK (Jing) 2016-0006; Beijing, China). Mice were housed in environmentally controlled cages (22 °C±2 °C, 45% ±15%) with a 12-hr light/dark cycle and free access to water and food. Mice were acclimatized for one week prior to experiments. Forty mice were randomly divided into the normal control (NC), corticosterone (Cor, 20 mg/kg), and therapy groups. The therapy groups included fluoxetine (Flu, 5 mg/kg), GS (3.33 g/kg), and Tax (100 mg/kg) groups. The Cor group was injected with corticosterone at a dose of 20 mg/kg for 21 d. Therapy groups were injected with corticosterone and were orally administered the indicated drugs for 21 d. NC and Cor groups were orally administered the same volume of distilled water.

***Sucrose preference tests***

Prior to sucrose preference tests, mice were adapted to sucrose solution for 72 hr. Two bottles of (1%, w/v) sucrose solution were placed in each cage for 24 hr, with one bottle of sucrose solution replaced with water. The location of the bottles were switched after 12 hr and mice were deprived of water and food for 24 h. Mice in each treatment group were housed in individual cages and provided free access to sucrose solution (1%, w/v) and water, After 12 hr, the volume (in ml) of the consumed solutions were recorded and sucrose preference was calculated.

***Sample preparation***

Urine samples were collected after 24 hr. Mice were sacrificed and blood samples were obtained from the eyeball. Samples were centrifuged at 3,000 rpm for 10 min, after which serum was isolated. Serum and urine samples were stored at −80 °C prior to analysis. Prior to metabolomic analysis, urine samples were thawed and aliquots were mixed with a three volumes of cold acetonitrile (4 °C). The mixture was vortexed for 1 min and centrifuged at 12,000 rpm for 10 min. Supernatants (2 µl) were injected for UPLC-Q/TOF-MS analysis.

Quality control (QC) sample preparation was as follows: QC samples were mixed with 50-μl aliquots of urine. Six consecutive injections of the QC sample were initially performed to avoid fluctuations. A further six samples were injected throughout the sequence.

***Assessment of serum corticosterone and 5-hydroxytryptamine levels***

Serum samples were thawed, and serum corticosterone and 5-hydroxytryptamine levels were assessed using ELISA kits according to the manufacturer’s protocols.

***UPLC-Q/TOF-MS analysis***

Metabolic profiling of urine samples was performed on an Ultra Performance Liquid Chromatography (UPLC) system (Dionex; UltiMate 3000 System, Thermo Scientific, USA) coupled to a Q/TOF mass spectrometer system equipped with an ESI ion source. Chromatographic separation was performed on an Acclaim™ RSLC 120 C18 column (2.1×100 mm, 2.2 µm) at a column temperature of 40 °C. The mobile phase consisted of 0.1% formic acid in water (A) and acetonitrile (B). Gradient elutions were programmed as followed: (0–3 min, 2%–10% B; 3–10 min, 10%–25% B; 10– 17 min, 25%–35% B; 17–19 min, 35%–60% B; and 19–20 min, 60%–98% B). The flow rate was 0.3 ml/min. MS properties were set as follows: scan range m/z, 60–1000 Da; positive mode capillary voltage, 3500 V; negative-mode capillary voltage, 3200 V; desolvation temperature, 230 °C; desolvation gas flow, 50 l/hr; and source power, 3.0 eV.

***Statistical analyses***

Raw UPLC-Q-TOF/MS data were calibrated and peak-aligned. Background noise was subtracted using Data Analysis 4.1 (Bruker, Germany). Processed data were converted to the bucket table using Profile Analysis 2.1 (Bruker, Germany) and were processed using SIMCA 13.0 (Umetrics AB, Sweden) for multivariate statistical analysis, including principal component analysis (PCA) and orthogonal partial least squares discriminant analysis (OPLS-DA). The variable importance in the projection (VIP) value of each variable in the OPLS-DA model was calculated. Differential metabolites were then obtained from the OPLS-DA model (VIP > 1). Differential metabolites were identified using the Human Metabolome Database (http://www.hmdb.ca/) and METLIN (https://metlin.scripps.edu/). The Kyoto Encyclopedia of Genes and Genomes (KEGG, http://www.genome.jp/kegg/) was used to identify metabolite-associated pathways, and a correlation network was used to visualize the disturbed pathways in the Cor groups. Heat maps of the metabolites were obtained through Multi Experiment Viewer (MeV) software 4.9.0, following the semi-quantitation of differential metabolites.

## Results

***Biochemical assessments***

**Figure 1:**
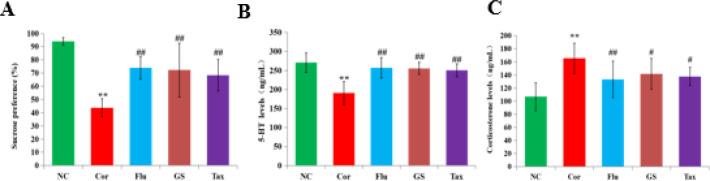
The effects of GS, Flu, and Tax on corticosterone. Sucrose consumption, and the serum levels of 5-hydroxytryptamine and corticosterone (A, B, C; mean±SD; n=8). ** *P*<0.001 vs NC group; ## *P*<0.01 vs Cor group; # *P*<0.01 vs Cor group

NC: normal control; Cor: corticosterone; Flu: fluoxetine; GS: Gleditsiae Spina; Tax: taxifolin

A significant reduction in sucrose consumption and serum 5-hydroxytryptamine occurred in Cor-induced depressed mice (Figures 1 A–C), while the serum levels of corticosterone were significantly increased compared to those of the NC group (P<0.01). Flu, GS, and Tax significantly improved these biochemical indexes (P<0.01).

***Global analysis of dynamic metabolic profiling***

**Figure 2:**
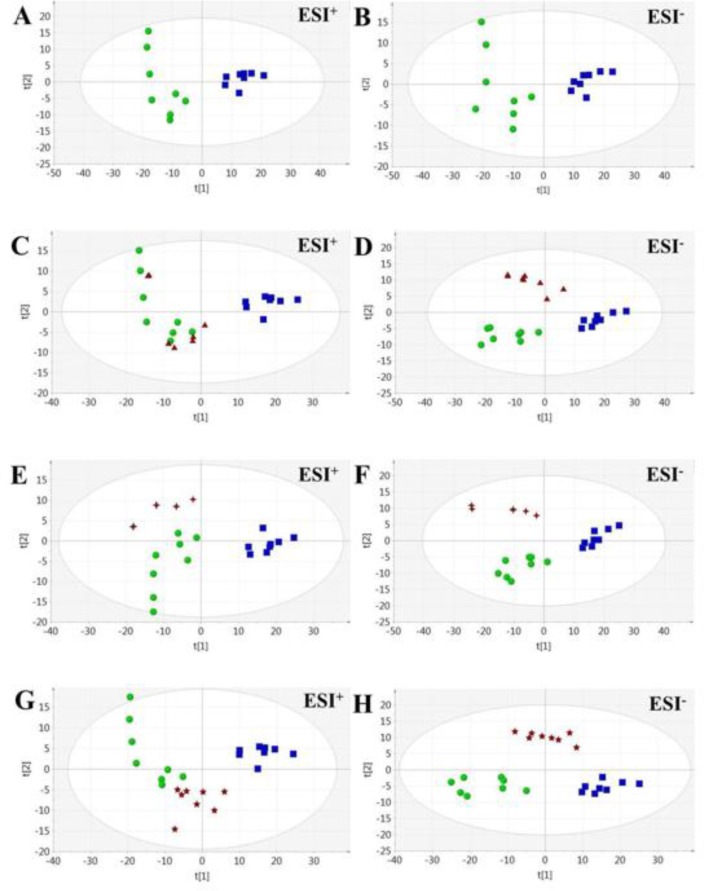
The effects of Flu, GS, and Tax on urinary metabolomic profiles of depressed mice

PCA analysis showed good separation between Cor and NC groups (NC *vs. *Cor: ESI^+^: R^2^X = 0.719 Q^2 ^= 0.509; ESI^-^: R^2^X = 0.804 Q^2 ^= 0.722), indicating that global metabolite fingerprints differed between NC and Cor rats (Figures 2A–B). Furthermore, the serum levels of corticosterone were significantly increased (*P*<0.01), while serum 5-hydroxytryptamine levels and sucrose consumption were significantly decreased (*P*<0.01), indicating successful establishment of our model (Figure 1). PCA score plots showed a clear separation of all groups, with both GS and Tax being closer to the NC group in terms of the first principal components. These results highlighted the antidepressant effects of the tested compounds (Figures 1C–H).

***Potential biomarkers***

**Figure 3:**
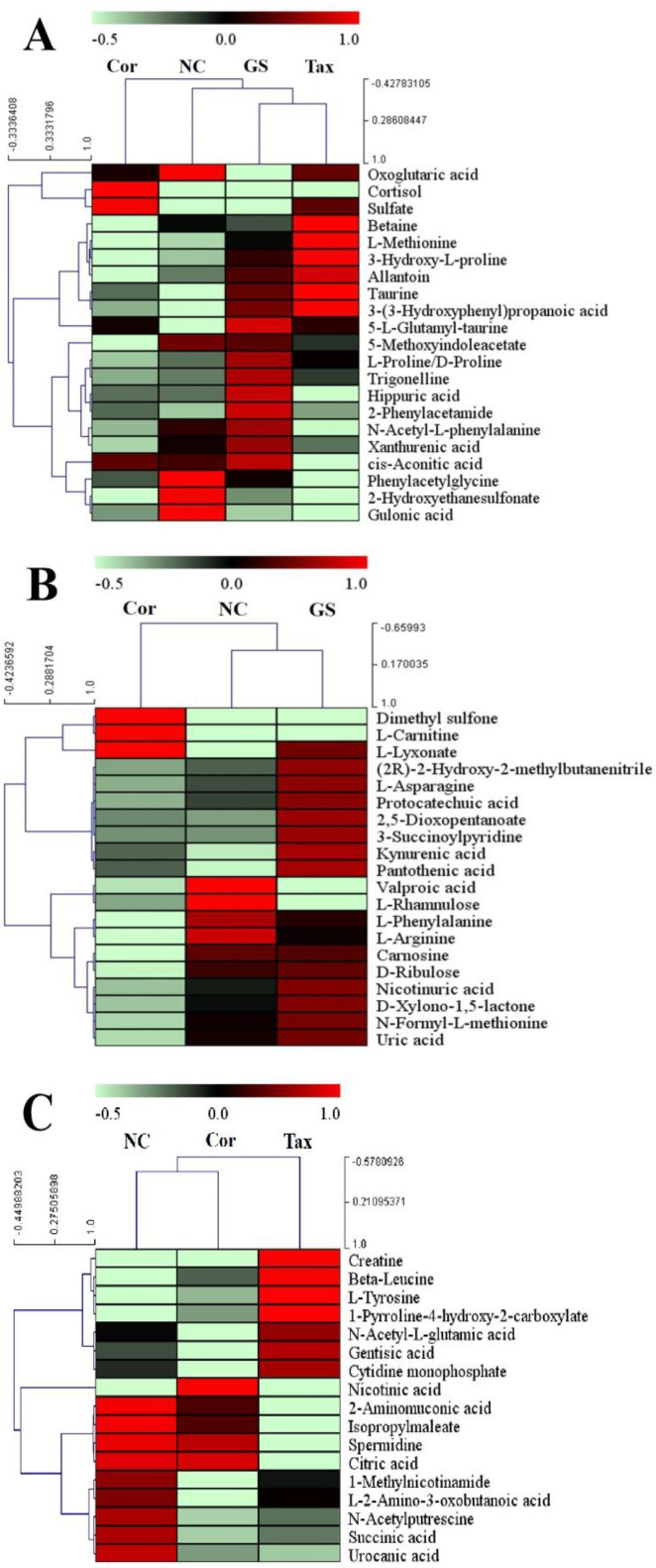
Heat maps of shared (A) and distinctive metabolites (B, C). The color scale ranges from red to green, which denotes upregulated or down regulated metabolites, respectively

**Figure 4:**
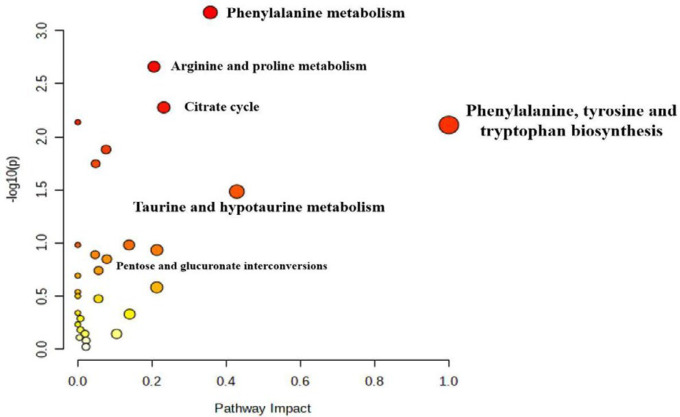
Metabolic pathway regulation analysis of the depressed groups after treatments with GS and Tax. Each point represents one metabolic pathway; the point size and shade of color positively correlates with the impact of the metabolic pathway

GS: Gleditsiae Spina; Tax: taxifolin

**Figure 5:**
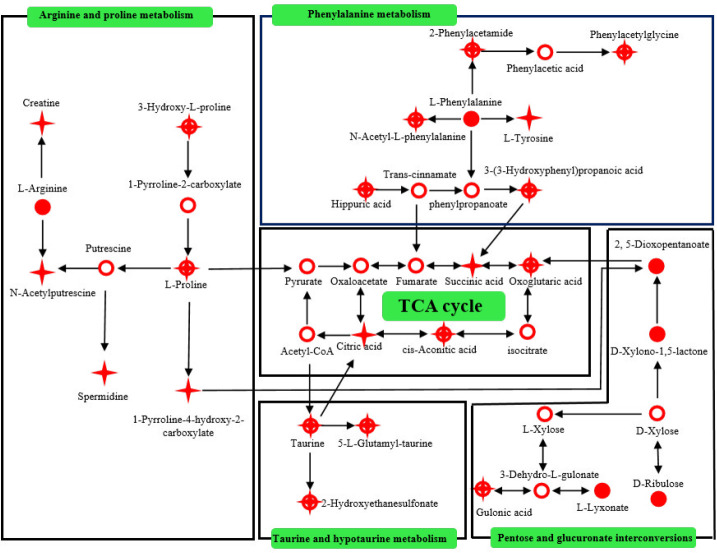
Metabolic pathway network affected by GS/Tax treatment

To compare the effects of GS and Tax, OPLS-DA score plots were constructed for both the positive and negative modes (Supplementary Figure 1). Obvious separations for GS *vs*. Cor and Tax *vs*. Cor were observed (ESI+: GS *vs.* Cor: R^2^X = 0.737, R^2^Y = 0.984, Q^2 ^= 0.958; Tax *vs*. Cor: R^2^X = 0.656, R^2^Y = 0.999, Q^2 ^= 0.946; ESI-: GS *vs*. Cor: R^2^X = 0.866, R^2^Y = 0.997, Q^2 ^= 0.98; Tax *vs*. Cor: R^2^X = 0.766, R^2^Y = 0.996, Q^2 ^= 0.974). To obtain the variable importance for the projections (VIP > 1), S-plot analyses were performed (Supplementary Figure 2). Potential biomarkers were identified through querying the Human Metabolome Database (http://www.hmdb.ca/) and Metlin (https://metlin.scripps.edu/). A total of 58 significantly differential urine metabolites were obtained as biomarkers related to the treatments of GS and Tax (Supplementary Table 1). To visualize the differences in relative values among all experimental groups, heat plots for the 58 differential metabolites were established using Mev (version 4.8.0, Figure 3). Pathway analysis combined with pathway enrichment analysis, hypergeometric overrepresentation tests, and topology analysis were conducted using MetaboAnakyst 5.0 (Table 1; Figure 4). The therapeutic effects of GS and Tax were found to be primarily associated with pentose and glucuronate interconversions, arginine and proline metabolism, phenylalanine metabolism, taurine and hypotaurine metabolism, and the citrate cycle. Then, metabolic networks were constructed to better understand the therapeutic effects of GS and Tax (Figure 5) using the KEGG database (http://www.kegg.jp/kegg/ pathway.html).

NC group; Cor group; Flu group; GS group and Tax group

NC: normal control; Cor: corticosterone; Flu: fluoxetine; GS: Gleditsiae Spina; Tax: taxifolin

metabolites in the GS group; metabolites in the Tax group; and shared metabolites between the GS and Tax groups

GS: Gleditsiae Spina; Tax: taxifolin

**Table 1:** Results of pathway analysis using MetaboAnalyst 5.0 for the depression groups after treatment with GS and Tax

Metabolic pathway	Total	Hits	Raw P-values	-log (p)	Holm adjust	FDR	Impact
Phenylalanine metabolism	12	4	0.00067552	3.1704	0.056743	0.056743	0.36
Arginine and proline metabolism	38	6	0.0021901	2.6595	0.18178	0.091985	0.21
Citrate cycle (TCA cycle)	20	4	0.0052932	2.2763	0.43404	0.13	0.23
Taurine and hypotaurine metabolism	8	2	0.032812	1.484	1	0.34452	0.43
Pentose and glucuronate interconversions	18	2	0.14176	0.84846	1	0.91596	0.08
Phenylalanine, tyrosine and tryptophan biosynthesis	4	2	0.0077382	2.1114	0.61905	0.13	1

Note: Raw p is the original* P-*value obtained by the analysis pathway. FDR and Holm’s procedures utilize the smallest *P*-value in order to reduced type-II error rates. Impact refers to the influence value of the pathway, which was obtained by topological analysis. The Total is the total number of compounds in the pathway; the Hits represents the actually matched number from the user uploaded data

GS: Gleditsiae Spina; Tax: taxifolin

## Discussion

In our present study, the depressed groups displayed reduced sucrose consumption, lower levels of serum 5-hydroxytryptamine, and higher levels of serum corticosterone, suggesting the successful establishment of our murine model of depression. GS and Tax markedly increased both the serum levels of 5-hydroxytryptamine and sucrose consumption, and reduced the serum levels of corticosterone, indicating that both GS and Tax exerted antidepressant effects. Furthermore, Tax was selected for metabolomic analysis, which verified that flavonoids mediated the antidepressant effects of GS.

***Pentose and glucuronate interconversions***

Fatigue is the most common symptom of depressed patients (20, 21). As an energy source, disorders in carbohydrate metabolism may be a cause of fatigue in depressed patients (22, 23). In the present study, the levels of intermediates formed during the interconversion of pentose and glucuronic acid—including 2,5-dioxopentanoate, D-xylonolactone, L-gulonate, L-lyxonate, and D-ribulose—were decreased in depressed mice, compared to those in the NC group (Supplementary Table 1). The levels of 2,5-dioxopentanoate, D-xylonolactone, L-gulonate, and D-ribulose were increased following the GS administration, indicating that GS mitigated the symptoms of depression through its regulation of pentose and glucuronate interconversions. Compared to those of GS, the effects of Tax were less pronounced, suggesting that other flavonoids may contribute to the beneficial effects of GS.

***Arginine and proline metabolism***

L-Arginine is the precursor of nitric oxide synthase (NOS), which catalyzes the formation of nitric oxide (NO) (24-26). As an important neurotransmitter, NO has modulatory effects on the central nervous system (CNS), including neurotransmission, neurogenesis, and synaptic plasticity (27-29). NO also relaxes blood vessels, improves circulation, and reduces lactic acid accumulation to exert its antifatigue effects (30). Compared to those in the NC group, the levels of L-arginine were decreased in the depression model group, which may contribute to fatigue in depressed patients (31, 32). Following the administration of GS, the levels of L-arginine were significantly increased. Arginine produces creatine, which is used in sports as an ergogenic aid (33-35). The levels of creatine increased following the administration of Tax, indicating that Tax may prevent fatigue in depressed patients.

Proline is a metabolite of glutamate (36). Glutamate is the major excitatory neurotransmitter in the mammalian nervous system (37, 38). Excess accumulation of glutamate at synapses between neurons leads to excitatory neurotoxicity, a major cause of depression (39, 40). As shown in Supplementary Table 1, low levels of proline were detected in the urine of depressed mice, indicating that the biosynthesis of proline from glutamate was inhibited. Following the administration of GS and Tax, proline levels were increased, indicating that GS and Tax reduced neurotoxicity. Taken together, these data demonstrate that, as a component of GS, Tax exhibits antidepressant activities through its ability to improve arginine and proline metabolism.

***Phenylalanine metabolism***

Phenylalanine is an essential amino acid. Phenylalanine can be converted into tyrosine by phenylalanine hydroxylase in the human kidney (41, 42). Catecholaminergic neurotransmitters—such as dopamine, norepinephrine, and adrenaline—are synthesized through the hydroxylation of tyrosine to L-dihydroxy-phenylalanine by tyrosine hydroxylase (43). Catecholaminergic levels show downward trends in depressed patients. In our present study, compared to those in the NC group, the levels of L-phenylalanine and L-tyrosine in the urine of the Cor group were significantly decreased. Following the administration of GS and Tax, GS upregulated the levels of L-phenylalanine, while Tax directly up-regulated the levels of L-tyrosine. Accordingly, both GS and Tax enhanced L-phenylalanine and L-tyrosine production, thereby alleviating the lack of catecholaminergic neurotransmitters in depressed mice.

***Taurine and hypotaurine metabolism***

Taurine is widely distributed as a free amino acid in the cerebral cortex (44), hippocampus (45), cerebellum (46), and bulbus olfactorius (47). Furthermore, taurine ameliorates depression-like behavior of mice through influencing the regulation of the hypothalamic-pituitary-adrenal axis to promote neurogenesis (48). In our present study, compared to those in the NC groups, the levels of taurine, 2-hydroxyethanesulfonate, and 5-L-glutamyl-taurine in the Cor group were decreased. Following the administration of GS and Tax, the levels of taurine in the urine were increased, while the metabolites of taurine, 2-hydroxyethanesulfonate, and 5-L-glutamyl-taurine showed a similar trend. These results demonstrate that Tax can be used as a substitute for GS to improve taurine and hypotaurine metabolism to exert antidepressant effects. Flavonoids may therefore represent the material basis of GS against depression.

***Citrate cycle***

The citrate cycle and oxidative phosphorylation are essential to energy metabolism, and glucose is metabolized into acetyl-CoA to participate in the citrate cycle (49, 50). A loss of glucose delivery leads to citrate attenuation and mitochondrial dysfunction, leading to neuronal death and irreversible cerebral injury (51). In the present study, carbohydrate metabolism was lower in depressed mice, which may lead to citrate-cycle-associated disorders. As an important product of the citrate cycle, the levels of oxoglutaric acid, cis-aconitic acid, citric acid, and succinic acid in the Cor group were significantly lower than those in the NC group. Furthermore, GS up-regulated the levels of cis-aconitic acid, while Tax increased the levels of succinic acid. In addition, GS/Tax increased the levels of oxoglutaric acid, highlighting their ability to overcome defects in the citrate cycle to exert antidepressant effects.

## Conclusion

We evaluated the antidepressant effects of GS and Tax via biochemical studies and metabolomic approaches. Five metabolic pathways were attributed to the possible antidepressant mechanisms of GS and Tax, including pentose and glucuronate interconversions, arginine and proline metabolism, phenylalanine metabolism, taurine and hypotaurine metabolism, and the citrate cycle. GS and Tax had similar effects on these five pathways and their metabolites. We therefore speculate that flavonoids form the basis of the antidepressant effects of GS. Collectively, our findings highlight the value of an integrated metabolomic approach to enhance our knowledge of the beneficial constituents of Chinese herbs.

## References

[1] Ahern E, Kinsella S, Semkovska M (2018). Clinical efficacy and economic evaluation of online cognitive behavioral therapy for major depressive disorder: a systematic review and meta-analysis. Expert Rev Pharmacoecon Outcomes Res.

[2] Kurhe Y, Mahesh R, Gupta D, Devadoss T (2014). QCM-4, a serotonergic type 3 receptor modulator attenuates depression co-morbid with obesity in mice: An approach based on behavioral and biochemical investigations. Eur J Pharmacol.

[3] Clarke TK, Obsteter J, Hall LS, Hayward C, Thomson PA, Smith BH (2017). Investigating shared aetiology between type 2 diabetes and major depressive disorder in a population based cohort. Am J Med Genet B Neuropsychiatr Genet.

[4] Schüle C (2007). Neuroendocrinological mechanisms of actions of antidepressant drugs. J Neuroendocrinol.

[5] Nabavi SM, Daglia M, Braidy N, Nabavi SF (2017). Natural products, micronutrients, and nutraceuticals for the treatment of depression: A short review. Nutr Neurosci.

[6] Huang KL, Lu WC, Wang YY, Hu GC, Lu CH, Lee WY (2014). Comparison of agomelatine and selective serotonin reuptake inhibitors/serotonin-norepinephrine reuptake inhibitors in major depressive disorder: A meta-analysis of head-to-head randomized clinical trials. Aust N Z J Psychiatry.

[7] Lee G, Bae H (2017). Therapeutic effects of phytochemicals and medicinal herbs on depression. Biomed Res Int.

[8] Wang D, Wang H, Gu L (2017). The antidepressant and cognitive improvement activities of the traditional chinese herb cistanche. Evid Based Complement Alternat Med.

[9] Yi JM, Kim J, Park JS, Lee J, Lee YJ, Hong JT (2015). In vivo anti-tumor effects of the ethanol extract of Gleditsia sinensis thorns and its active constituent. Cytochalasin H Biol Pharm Bull.

[10] Fang LH, Wang RP, Hu SY, Teng YH, Xie WB (2015). The effect of tou nong san on transplanted tumor growth in nude mice. Evid Based Complement Alternat Med.

[11] Shin TY (2010). The extract of Gleditsiae spina inhibits mast cell-mediated allergic reactions through the inhibition of histamine release and inflammatory cytokine production. Nat Prod Res.

[12] Lee SJ, Park SS, Kim WJ, Moon S (2012). Gleditsia sinensis thorn extract inhibits proliferation and TNF-ɑ-induced MMP-9 expression in vascular smooth muscle cells. Am J Chin Med.

[13] Li J, Jiang K, Wang LJ, Yin G, Wang J, Wang Y (2018). HPLC-MS/MS determination of flavonoids in Gleditsiae spina for its quality assessment. Sep Sci.

[14] Yu J, Zhao L, Sun X, Sun C, Wang X (2020). Application of choline chloride deep eutectic solvents and high-speed counter-current chromatography to the extraction and purification of flavonoids from the thorns of Gleditsia sinensis Lam. Phytochem Anal.

[15] Saito S, Yamamoto Y, Maki T, Hattori Y, Ito H, Mizuno K (2017). Taxifolin inhibits amyloid-β oligomer formation and fully restores vascular integrity and memory in cerebral amyloid angiopathy. Acta Neuropathol Commun.

[16] Kuang H, Tang Z, Zhang C, Wang Z, Li W, Yang C (2017). Taxifolin activates the Nrf2 anti-oxidative stress pathway in mouse skin epidermal JB6 P+ cells through epigenetic modifications. Int J Mol Sci.

[17] Park SY, Kim HY, Park HJ, Shin HK, Hong KW, Kim CD (2016). Concurrent treatment with taxifolin and cilostazol on the lowering of β-amyloid accumulation and neurotoxicity via the suppression of P-JAK2/P-STAT3/NF-κB/BACE1 signaling pathways. PLoS One.

[18] Inoue T, Saito S, Tanaka M, Yamakage H, Kusakabe T, Shimatsu A (2019). Pleiotropic neuroprotective effects of taxifolin in cerebral amyloid angiopathy. Proc Natl Acad Sci USA.

[19] Gunesch S, Soriano-Castell D, Lamer S, Schlosser A, Maher P, Decker M (2020). Development and application of a chemical probe based on a neuroprotective flavonoid hybrid for target identification using activity-based protein profiling. ACS Chem Neurosci.

[20] Liu CC, Wu YF, Feng GM, Gao XX, Zhou YZ, Hou WJ (2015). Plasma-metabolite-biomarkers for the therapeutic response in depressed patients by the traditional Chinese medicine formula Xiaoyaosan: A (1)H NMR-based metabolomics approach. Affect Disord.

[21] Nyer M, Mischoulon D, Alpert JE, Holt DJ, Brill CD, Yeung A (2015). College students with depressive symptoms with and without fatigue: Differences in functioning, suicidality, anxiety, and depressive severity. Ann Clin Psychiatry.

[22] Enko D, Wagner H, Kriegshäuser G, Brandmayr W, Halwachs-Baumann G, Schnedl WJ (2018). Assessment of tryptophan metabolism and signs of depression in individuals with carbohydrate malabsorption. Psychiatry Res.

[23] Wurtman RJ, Wurtman JJ (1995). Brain serotonin, carbohydrate-craving, obesity and depression. Obes Res.

[24] Umar S, van der Laarse A (2010). Nitric oxide and nitric oxide synthase isoforms in the normal, hypertrophic, and failing heart. Mol Cell Biochem.

[25] Böger RH (2014). The pharmacodynamics of L-arginine. Altern Ther Health Med.

[26] He HY, Henderson AC, Du YL, Ryan KS (2019). Two-enzyme pathway links l-arginine to nitric oxide in N-nitroso biosynthesis. J Am Chem Soc.

[27] Chong CM, Ai N, Ke M, Tan Y, Huang Z, Li Y (2018). Roles of nitric oxide synthase isoforms in neurogenesis. Mol Neurobiol.

[28] Joca SRL, Sartim AG, Roncalho AL, Diniz CFA, Wegener G (2019). Nitric oxide signalling and antidepressant action revisited. Cell Tissue Res.

[29] Sanders KM, Ward SM (2019). Nitric oxide and its role as a non-adrenergic, non-cholinergic inhibitory neurotransmitter in the gastrointestinal tract. Br J Pharmacol.

[30] Zhang Q, Deng Y, Zhang W, Liu Y, Zha D (2016). Drag-reducing polymers increase exercise tolerance in an ischemic hind-limb rat model. Vascular.

[31] Hess S, Baker G, Gyenes G, Tsuyuki R, Newman S, Melledo LJM (2017). Decreased serum L-arginine and L-citrulline levels in major depression. Psychopharmacology.

[32] Ali-Sisto T, Tolmunen T, Viinamäki H, Mäntyselkä P, Valkonen-Korhonen M, Koivumaa-Honkanen H (2018). Global arginine bioavailability ratio is decreased in patients with major depressive disorder. J Affect Disord.

[33] Markus W, Rima KD (2000). Creatine and creatinine metabolism. Physiol Rev.

[34] Ji L, Zhao X, Zhang B, Kang L, Song W, Zhao B (2019). Slc6a8-mediated creatine uptake and accumulation reprogram macrophage polarization via regulating cytokine responses. Immunity.

[35] Tarnopolsky MA (2010). Caffeine and creatine use in sport. Ann Nutr Metab.

[36] Cappelletti P, Tallarita E, Rabattoni V, Campomenosi P, Sacchi S, Pollegioni L (2018). Proline oxidase controls proline, glutamate, and glutamine cellular concentrations in a U87 glioblastoma cell line. PLoS One.

[37] Hull J, Usmari Moraes M, Brookes E, Love S, Conway ME (2018). Distribution of the branched-chain ɑ-ketoacid dehydrogenase complex E1ɑ subunit and glutamate dehydrogenase in the human brain and their role in neuro-metabolism. Neurochem Int.

[38] Zhou Y, Danbolt NC (2014). Glutamate as a neurotransmitter in the healthy brain. J Neural Transm (Vienna).

[39] Fouad IA, Sharaf NM, Abdelghany RM, Dine ESNSE (2018). Neuromodulatory effect of thymoquinone in attenuating glutamate-mediated neurotoxicity targeting the amyloidogenic and apoptotic pathways. Front Neurol.

[40] Lau A, Tymianski M (2010). Glutamate receptors, neurotoxicity and neurodegeneration. Pflugers Arch.

[41] Ge Y, Borne E, Stewart S, Hansen MR, Arturo EC, Jaffe EK (2018). Simulations of the regulatory ACT domain of human phenylalanine hydroxylase (PAH) unveil its mechanism of phenylalanine binding. Biol Chem.

[42] Mitchell JJ, Trakadis YJ, Scriver CR (2011). Phenylalanine hydroxylase deficiency. Genet Med.

[43] Cieńska M, Labus K, Lewańczuk M, Koźlecki T, Liesiene J, Bryjak J (2016). Effective L-tyrosine hydroxylation by native and immobilized tyrosinase. PLoS One.

[44] Paulucio D, Terra A, Santos CG, Cagy M, Velasques B, Ribeiro P (2018). Acute effect of Ethanol and Taurine on frontal cortex absolute beta power before and after exercise. PLoS One.

[45] Hansen AW, Almeida FB, Bandiera S, Pulcinelli RR, Caletti G, Agnes G (2020). Correlations between subunits of GABAA and NMDA receptors after chronic alcohol treatment or withdrawal, and the effect of taurine in the hippocampus of rats. Alcohol.

[46] Wei L, Xue R, Zhang P, Wu Y, Li X, Pei F (2015). 1H NMR-Based metabolomics and neurotoxicity study of cerebrum and cerebellum in rats treated with cinnabar, a traditional chinese medicine. OMICS.

[47] Belluzzi O, Puopolo M, Benedusi M, Kratskin I (2004). Selective neuroinhibitory effects of taurine in slices of rat main olfactory bulb. Neuroscience.

[48] Wu GF, Ren S, Tang RY, Xu C, Zhou JQ, Lin SM (2017). Antidepressant effect of taurine in chronic unpredictable mild stress-induced depressive rats. Sci Rep.

[49] Kori Y, Sidoli S, Yuan ZF, Lund PJ, Zhao X, Garcia BA (2017). Proteome-wide acetylation dynamics in human cells. Sci Rep.

[50] Lauterbach MA, Hanke JE, Serefidou M, Mangan MSJ, Kolbe CC, Hess T (2019). Toll-like receptor signaling rewires macrophage metabolism and promotes histone acetylation via atp-citrate lyase. Immunity.

[51] Suh SW, Hamby AM, Gum ET, Shin BS, Won SJ, Sheline CT (2018). Sequential release of nitric oxide, zinc, and superoxide in hypoglycemic neuronal death. J Cereb Blood Flow Metab.

